# Autologous Cells for Kidney Bioengineering

**DOI:** 10.1007/s40472-016-0107-8

**Published:** 2016-06-09

**Authors:** Bettina Wilm, Riccardo Tamburrini, Giuseppe Orlando, Patricia Murray

**Affiliations:** 1 Institute of Translational Medicine, Centre for Preclinical Imaging, University of Liverpool, Crown Street, Liverpool, L69 3BX UK; 2 Department of Surgery, Section of Transplantation, Wake Forest School of Medicine,Wake Forest Baptist Hospital, Medical Center Blvd, Winston Salem, NC 27157 USA

**Keywords:** Induced pluripotent stem cells, Renal progenitor cells, Kidney organoids, Decellularisation, Kidney scaffolds, Bioprinting

## Abstract

Worldwide, increasing numbers of patients are developing end-stage renal disease, and at present, the only treatment options are dialysis or kidney transplantation. Dialysis is associated with increased morbidity and mortality, poor life quality and high economic costs. Transplantation is by far the better option, but there are insufficient numbers of donor kidneys available. Therefore, there is an urgent need to explore alternative approaches. In this review, we discuss how this problem could potentially be addressed by using autologous cells and appropriate scaffolds to develop ‘bioengineered’ kidneys for transplantation. In particular, we will highlight recent breakthroughs in pluripotent stem cell biology that have led to the development of autologous renal progenitor cells capable of differentiating to all renal cell types and will discuss how these cells could be combined with appropriate scaffolds to develop a bioengineered kidney.

## Introduction

Over recent years, there has been an increasing interest in developing stem cell-based regenerative medicine therapies for patients with kidney disease. Stem cell therapies are already showing great promise in rodent models of acute and chronic kidney disease [1], and several clinical trials are now underway to assess the safety and efficacy of these novel therapies in humans with kidney disease (see Table 1). It should be noted, however, that while stem cell therapies could be useful for ameliorating acute or chronic renal injury, the consensus view is that they would be of little benefit in the context of end-stage renal disease (ESRD). The best treatment option for ESRD is kidney transplantation, but the shortage of donor kidneys means that most patients do not get offered a transplant, a situation which has stimulated efforts to develop ‘bioengineered’ kidneys. Whilst challenging, advances in biomaterials research and stem cell biology, including cellular reprogramming technologies, means that bioengineered kidneys for patients with ESRD could be possible in the future. For instance, in 2013, a bioengineered rat kidney was constructed by seeding rat neonatal kidney cells and human umbilical cord endothelial cells on a decellularised adult rat kidney scaffold [2••]. Importantly, these synthetic kidneys showed some evidence of functionality and could produce ‘rudimentary’ urine in rat hosts [2••]. For human patients, the ideal components of a bioengineered kidney would be autologous stem cells and non-immunogenic biomaterial scaffolds, thus avoiding immune rejection and/or life-long treatment with immunosuppressants. In this review, we will discuss current progress towards the development of bioengineered kidneys, with particular focus on the following key issues: (i) the optimal source of autologous stem cells, (ii) bioengineering strategies and (iii) safety aspects.

**Table 1:** Clinical trials using stem cell therapies

Clinical trial name	Clinical trial identifier	Purpose	Status	Sponsor	Estimated study completion date
Pilot Feasibility Study of Combined Kidney and Hematopoietic Stem Cell Transplantation to Cure End-stage Renal Disease	NCT02176434	This pilot study of combined kidney and hematopoietic stem cell transplantation attempts to establish a protocol to induce immunological tolerance as a new strategy to prevent renal graft rejection. If successful, this strategy would restore renal function, while avoiding the risks associated with long-term standard anti-rejection therapy, and would represent the first option to cure end-stage renal disease.	Recruiting	University of Zurich	August 2018
Mesenchymal Stem Cells Transplantation in Patients With Chronic Renal Failure Due to Polycystic Kidney Disease	NCT02166489	This study was designed to provide confirmation of safety of mesenchymal stem cells (MSCs) therapy in chronic renal failure due to autosomal dominant polycystic kidney disease (ADPKD).	Completed	Royan Institute, Tehran	January 2016
Using Donor Stem Cells and Alemtuzumab to Prevent Organ Rejection in Kidney Transplant Patients	NCT00183248	This study will evaluate treatment of kidney transplant recipients with alemtuzumab and other immune system suppressing medications with or without infusions of bone marrow stem cells from the kidney donor. The purpose of this study is to find out which strategy is more effective in preventing organ rejection and maintaining patient health.	Completed	University of Miami	November 2009
Safety and Efficacy of Autologous Bone Marrow Stem Cells for Treating Chronic Renal Failure	NCT01152411	To evaluate the safety and efficacy (to know / observe for Proof of concept in five Indian patients) if any, of autologous bone marrow derived stem cells injected into the Renal Artery in five (initially five patients, can be increased to ten patients after observing the initial results) patients with Chronic Renal Failure	Unknown	International Stem Cell Services Limited	Unknown
Induction of Donor Specific Tolerance in Recipients of Living Kidney Allografts by Donor FCRx Infusion	NCT00497926	Use of a combination of an Enriched Hematopoetic Stem Cell Infusion and kidney transplantation from the same donor to try to avoid the need for long-term anti-rejection drug therapy. The desired result of this study is to allow the body to develop “tolerance” to the transplanted kidney.	Recruiting	University of Louisville	March 2030
Effect of BM-MSCs in DCD Kidney Transplantation	NCT02561767	To determine the efficacy and safety of allogeneic bone marrow-derived mesenchymal stem cells in kidney transplantation from Chinese donation after citizen’s death (DCD).	Not yet Opened for Recruitment	Sun Yat-Sen University	October 2017
Induction of Donor Specific Tolerance in Recipients of Live Donor Kidney Allografts by Donor Stem Cell Infusion	NCT00498160	Induction of Donor Specific Tolerance in Recipients of Kidney Allografts by Donor Bone Marrow Cell Infusion (Deceased Donors) and Induction of Donor Specific Tolerance in Recipients of Live Donor Kidney Allografts by Donor Stem Cell Infusion	Current	University of Louisville	December 2024
Mesenchymal Stem Cells After Renal or Liver Transplantation	NCT01429038	To evaluate the safety and tolerability of MSC administration after liver or kidney transplantation.	Recruiting	University Hospital of Liege	February 2017
Autologous Neo-Kidney Augment (NKA) in Patients With Type 2 Diabetes and Chronic Kidney Disease (CKD) (RMCL-CL001)	NCT02525263	A Phase II, Open-Label Safety and Efficacy Study of an Autologous Neo-Kidney Augment (NKA) in Patients With Type 2 Diabetes and Chronic Kidney Disease (RMTX-CL001). NKA is made from expanded autologous selected renal cells (SRC) obtained from the patient’s kidney biopsy. All enrolled subjects will be treated with up to two injections of NKA at least 6 months apart.	Not yet Opened for Recruitment	RegenMed (Cayman) Ltd.	January 2018
Induction Therapy With Autologous Mesenchymal Stem Cells for Kidney Allografts	NCT00658073	To evaluate autologous MSCs as an alternative for antibody induction therapy in renal transplantation	Completed	Fuzhou General Hospital	October 2010
Mesenchymal Stem Cell Transplantation in the Treatment of Chronic Allograft Nephropathy	NCT00659620	The purpose of this study is to find out MSC is more effective in preventing organ rejection and maintaining kidney function.	Completed	Fuzhou General Hospital	May 2010
Tolerance Induction in Living Donor Kidney Transplantation With Hematopoietic Stem Cell Transplantation	NCT02199301	To evaluate the Tolerance induction in KT recipients with donor hematopoietic stem cell transplantation (HSCT).	Recruiting	Samsung Medical Center	December 2017
MSC for Occlusive Disease of the Kidney	NCT01840540	To determine the safety and toxicity of intra-arterial infused autologous adipose derived mesenchymal stromal (stem) cells in patients with vascular occlusive disease of the kidney.	Opened	Mayo Clinic	April 2017
Autologous Bone Marrow Derived Mesenchymal Stromal Cells (BM-MSCs) in Patients With Chronic Kidney Disease (CKD)	NCT02195323	To provide confirmation of safety of mesenchymal stem cells (MSCs) therapy in chronic kidney disease (CKD).	Completed	Royan Institute	January 2016
Kidney and Blood Stem Cell Transplantation That Eliminates Requirement for Immunosuppressive Drugs	NCT00319657	To determine if blood stem cells injected after kidney transplantation will change the immune system such that immunosuppressive drugs can be completely withdrawn. Patients must have a healthy, completely human leukocyte antigen (HLA)-matched brother or sister as the organ and stem cell donor.	Recruiting	Stanford University	July 2016
Mesenchymal Stem Cells In Cisplatin-Induced Acute Renal Failure In Patients With Solid Organ Cancers (CIS/MSC08)	NCT01275612	To test the feasibility and safety of systemic infusion of donor ex-vivo expanded Mesenchymal Stem Cells to repair the kidney and improve function in patients with solid organ cancers who develop acute renal failure after chemotherapy with cisplatin.	Recruiting	Mario Negri Institute for Pharmacological Research	March 2017
Stem Cell Therapy for Patients With Focal Segmental Glomerulosclerosis (STEFOG)	NCT02693366	To analyze the safety, renal function, metabolic disorders and quality of life data in patients with focal segmental glomerulosclerosis treated with endovascular infusion of bone marrow derived mononuclear cells.	Recruiting	Universidade Federal do Rio de Janeiro	June 2017
Effect of BM-MSCs on Early Graft Function Recovery After DCD Kidney Transplant.	NCT02563366	This study is designed to investigate whether allogeneic bone marrow-derived mesenchymal stem cells (BM-MSCs) can promote function recovery in patients with poor early graft function after kidney transplantation from Chinese Donation after Citizen Death (DCD).	Not yet Opened for Recruitment	Sun Yat-Sen University	December 2017
Mesenchymal Stem Cells Under Basiliximab/Low Dose RATG to Induce Renal Transplant Tolerance	NCT00752479	To define the safety and biological/mechanistic effect of the systemic intravenous infusion of syngeneic ex-vivo expanded MSCs in living-related kidney transplant recipients (one or two HLA haplotype mismatches) under basiliximab/low-dose RATG induction therapy and maintenance immunosuppressive drugs with the ultimate objective to test the feasibility of safely achieving graft tolerance in a subsequent efficacy pilot study.	Terminated	Mario Negri Institute for Pharmacological Research	December 2013
Safety and Efficacy of BMMNC in Patients With Chronic Renal Failure	NCT01876017	Single center trial to check the safety and efficacy of Autologous Bone Marrow derived Mono Nuclear Stem Cell (BMMNCs) for the patient with CRF	Recruiting	Chaitanya Hospital, Pune	December 2016
Study to Assess the Safety and Effects of Autologous Adipose-Derived Stromal Cells Delivered in Patients With Renal Failure	NCT01453816	An Open-label, Non-Randomised, Multi-Center Study to Assess the Safety and Effects of Autologous Adipose-Derived Stromal Cells Delivered Into the Renal Artery and Intravenously in Patients With Renal Failure	Unknown	Ageless Regenerative Institute	June 2015
Hypoxia and Inflammatory Injury in Human Renovascular Hypertension	NCT02266394	To determine if the MSC infusion prior to percutaneous transluminal renal angioplasty with stenting (PTRA) further enhances changes in single kidney blood flow and restoration of kidney function, as well as to assess the relationship between MSC dose and measures of kidney function.	Recruiting	Mayo Clinic	March 2019
To Elucidate the Effect of Mesenchymal Stem Cells on the T Cell Repertoire of the Kidney Transplant Patients	NCT02409940	Aim To investigate effect of MSCs on immune cell repertoire in a donor specific mediated response.	Recruiting	Postgraduate Institute of Medical Education and Research	December 2016
Mesenchymal Stem Cell Transplantation in the Treatment of Chronic Allograft Nephropathy	NCT00659620	Mesenchymal Stem Cell (MSC) has been shown to have immunosuppressive and repairing properties. the investigators will infuse expanded MSC into patients who develop Chronic Allograft Nephropathy. The purpose of this study is to find out MSC is more effective in preventing organ rejection and maintaining kidney function	Unknown	Fuzhou General Hospital	May 2010

Stem Cells and Kidney Disease – Clinical Trials; Source: www.ClinicalTrials.gov

## Sourcing Autologous Cells with Renal Differentiation Potential

The kidney is one of the most complex organs in the human body, consisting of more than 26 different cell types [3]. Many studies have analysed the potential of autologous cells for treating kidney disease, both in preclinical models and in the clinic. There has been particular focus on the use of cells that either have their origin in the kidney or on cells of non-renal origin that can nevertheless generate specialised renal cells and can be easily sourced from the patient. Here, we give a brief overview of the most well-studied autologous sources, which include kidney-derived cells (KCs), mesenchymal stromal/stem cells (MSCs), adipose-derived regenerative cells (ADRCs) and induced pluripotent stem cells (iPSCs).

## Adult Kidney Cells

A number of different approaches have been followed to identify, isolate and characterise stem or progenitor cells from human kidney biopsies, typically by investigating clonogenicity, expression of stem cell markers, differentiation potential and ability to ameliorate kidney injury in vivo following administration into rodent disease models [4–10]. One of the key tools has been the chimeric embryonic kidney rudiment assay developed by Unbekandt and Davies and its modified versions [11–14]. With this in vitro approach, the potential of the stem/progenitor cells to undergo renal differentiation can be assessed by mixing the cells with dissociated embryonic mouse kidney cells, which are then re-aggregated to form a chimeric rudiment. Using this approach, our group was able to show that kidney-derived stem cells isolated from newborn mice have the potential to integrate into embryonic kidney rudiments and contribute to developing nephron structures and glomeruli [15].

In human kidneys, NCAM, Tra-1-60 and CD133 have been identified as putative stem/progenitor cell markers [16–21]. In vitro characterisation assays suggested that CD133^+^ cells have a range of stem cell properties, including clonogenicity, self-renewal and the potential to differentiate along the renal, endothelial, adipogenic and osteogenic lineages [18–21]. Furthermore, administration of the cells into the tail vein of mice with rhabdomyolysis-induced acute tubular injury, or adriamycin-induced glomerular injury, resulted in amelioration of histological damage and improved function [18, 20, 22, 23]. In these studies, the authors provided evidence that some of the CD133^+^ KCs have the potential to integrate into the affected renal structures, contributing to their repair.

An advantage of autologous CD133^+^ KCs is that they are already committed to the renal lineage and would therefore be expected to differentiate into specialised renal cells quite readily. A major drawback, however, is that the number of healthy KCs that could be retrieved from a renal biopsy from a patient with ESRD would probably be too small to permit adequate expansion in vitro; this is because the CD133^+^ KCs change their phenotype and become senescent after ∼7 passages, thus limiting their expansion capacity [18, 24, 25]. Furthermore, there is no evidence that CD133^+^ KCs can generate all of the 26 different cell types in the kidney, so it is unlikely that they could be used to generate a bioengineered kidney.

## MSCs

MSCs can contribute to the regeneration and repair of various organs. However, although it has been reported that MSCs can generate specialised renal cells [26, 27], more recent studies have shown that the regenerative effects of MSCs in various rodent kidney injury models are mediated by paracrine factors, including growth factors and extracellular vesicles [28–30, 31•, 32–34], which can modulate the immune system and suppress inflammation. For instance, a recent study has shown that following intravenous injection of MSCs into a rhabdomyolysis model of tubular injury, despite significant improvement in renal histology and function, most cells were located in the lungs or injured muscle, and none were present in the kidney [31•]. Even following direct administration into the kidney via the renal artery, MSCs were only transiently located within the glomerular capillaries or interstitium and did not differentiate into renal cells [32, 35–38]. Moreover, MSCs that did persist in the kidney appeared to differentiate into adipocytes within the glomeruli [39] and had an adverse effect on renal health. Taken together, these studies show that the therapeutic effects of MSCs are mediated by paracrine or even endocrine factors, which probably improve renal health by modulating the immune system. Consequently, MSCs would be of little use in the development of a bioengineered kidney.

## ADRCs

ADRCs have recently become of interest as regenerative medicine therapies, not only because of their accessibility, but also due to their efficacy in repairing tissue damage, including ischaemia-induced injuries [40–44]. Recently, Cytori have developed a method for processing ADRCs under good laboratory practice (GLP) compliance by dissociating the adipose tissue and enriching the ADRCs in a functionally closed system using proprietary reagents [45, 46]. However, similarly to MSCs, ADRCs appear to ameliorate injury by paracrine factors rather than by differentiating to replace damaged tissue [40] and would be unable to generate the different types of renal cells required to make a bioengineered kidney.

Thus, although therapeutic efficacy has been demonstrated for KCs, MSCs and ADRCs in rodent kidney injury models, there is no evidence that these cells can permanently integrate into injured kidneys or differentiate in situ to replace all types of damaged cells renal cells. Of note, for ADRCs or MSCs, only a limited capacity to differentiate into epithelial cells in vitro has been reported [47, 48]. This was supported by our own work using the chimeric embryonic kidney rudiment assay, in which both human and murine MSCs demonstrated not only failure to integrate and contribute to the development of renal structures, but also negatively affected the formation of nephron structures [49]. These observations indicate that while MSCs and ADRCs could be effective autologous therapies for acute or even early stage chronic kidney disease, they would have no place in renal bioengineering strategies to treat ESRD patients. Adult KCs appear to have at least some renal differentiation potential in vitro, albeit limited, but autologous sourcing would be problematic, especially for patients with ESRD where very little healthy renal tissue remains.

## iPSCs

Several studies have previously shown that murine embryonic stem cell (ESC)-derived mesodermal cells can be directed to differentiate into a range of renal cell types. This was achieved using various techniques, including co-culture methods with embryonic spinal cord, the kidney rudiment assay, or after injection ex vivo or in vivo into newborn mouse kidneys [13, 50–53]. Although encouraging, a major drawback with ESCs is that they are not autologous and would therefore induce an immune response if incorporated into a bioengineered kidney. However, the development by Yamanaka and colleagues of a strategy to reprogramme adult cells into ESC-like induced pluripotent stem cells (iPSCs) [54] means that autologous pluripotent stem cells are now available for personalised cell therapies. The original cocktail of Yamanaka factors, which consisted of Oct3/4, Sox2, Klf4 and c-myc, has since been optimised to replace the two oncogenes, c-myc and Klf4 [55], thus making the iPSCs less tumourigenic. Furthermore, the use of ‘non-integrating’ methods to introduce the reprogramming factors has circumvented the need for lenti- or retroviral vectors [56], which pose safety issues due to the fact that they integrate into the genome and can induce oncogenic transformation [57].

**Fig. 1:**
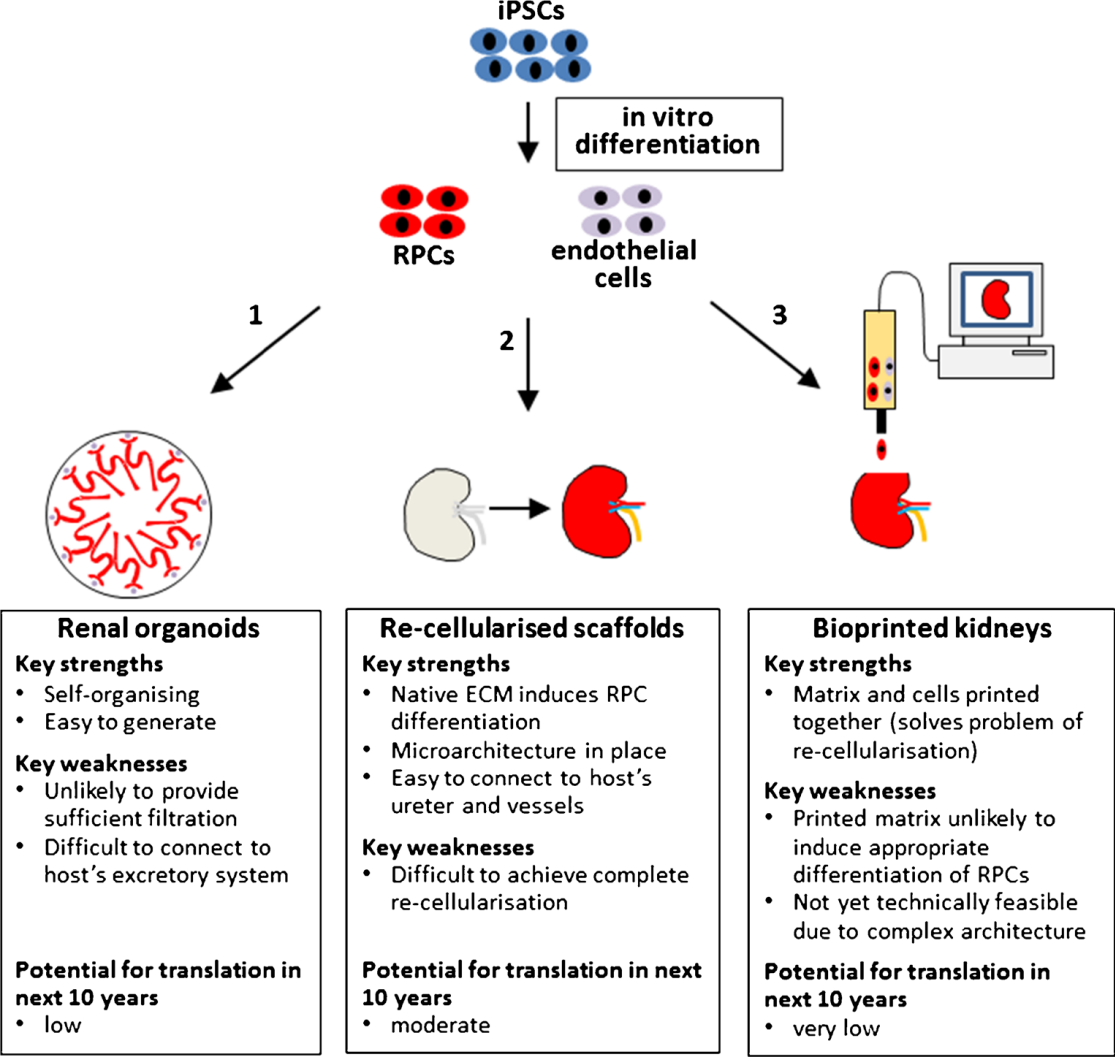
Schematic diagram showing 3 potential methods for making bioengineered kidneys using autologous cells. *1* iPSC-derived RPCs and endothelial cells self-organise in vitro to generate renal organoids. *2* iPSC-derived RPCs and endothelial cells are introduced into decellularised human or pig kidneys via the renal artery (endothelial cells) and ureter (RPCs). *3* iPSC-derived RPCs, endothelial cells and an appropriate matrix are printed according to a computer-generated organ ‘blueprint’

Over the last few years, a number of groups have been able to develop protocols to direct the differentiation of iPSCs to nephron progenitor cells (capable of generating cells of the nephron; also known as ‘metanephric mesenchyme’) and renal progenitor cells (RPCs) (capable of generating cells of the nephron, collecting tubules and interstitium). Seminal work in this field came from the Nishinakamura group, who, using information gleaned from the mouse embryo, designed a 3-stage differentiation protocol for directing the differentiation of ESCs or iPSCs to nephron progenitors [58••]. This was achieved by incubating ESCs or iPSCs with various growth factors, including Activin, Bmp4, FGF9 and the Wnt agonist CHIR99021, at specific time points to mimic the temporal regulation of mesoderm differentiation in vivo. Apart from Nishinakamura, various other groups have described protocols for directing the differentiation of iPSCs into the renal lineage [58••, 59–62, 63••, 64••, 65, 66, 67•], with recent studies by the Little and Bonventre groups showing that iPSC-derived RPCs can self-organise into kidney organoids containing glomerular and tubular structures with evidence of transport function and endothelial cell integration [63••, 64••, 68]. These studies present an exciting breakthrough in the field because for the first time, they show that all cells of the kidney can be generated from an autologous cell source, potentially opening the door to the development of bioengineered kidneys (Fig. 1). In the next section, we will discuss three strategies whereby iPSC-derived RPCs could be used for this purpose.

## Bioengineering Strategies

Here, we will focus on the following three strategies that have potential for developing bioengineered kidneys in the future: (i) self-organisation of RPCs to generate renal organoids, (ii) seeding of RPCs into decellularised kidney scaffolds and (iii) 3D bioprinting of RPCs and synthetic matrices.

## RPC-Derived Organoids

Since the 1990s, various groups have explored the possibility of transplanting kidney rudiments derived from rodent, pig and human foetal kidneys into adult hosts. In most cases, the rudiments showed some evidence of growth and functionality, irrespective of whether they were transplanted under the kidney capsule [69, 70], into the kidney parenchyma [71], near the abdominal aorta [72] or into the omentum [70, 73, 74]. However, there are several technical problems with rudiment transplantation that would prevent this approach from being used in the clinic. Firstly, the rudiments would be non-autologous and therefore immunogenic. Secondly, in these early studies, the rudiments did not connect to the host’s ureter, leading in some cases to the development of hydronephrosis. Thirdly, although the rudiments grew in their new hosts, they did not mature beyond a neonatal stage and their filtering capacity was only equivalent to 2 % of that of an adult kidney [72, 75].

With the advancements in generating iPSC-derived RPCs, the first of these problems could now be overcome. As discussed above, under the appropriate culture conditions, iPSC-derived RPCs can give rise to both metanephric mesenchyme (the nephron progenitors) and ureteric bud (the progenitors of the collecting tubules and ureter) [63••, 64••]. Remarkably, it was shown that these two primordial cell types could differentiate appropriately in vitro and self-organise to form 3D organoids comprising nephrons complete with glomeruli, proximal and distal tubules and loops of Henle, which were associated with ureteric bud-derived collecting tubules. These organoids also had iPSC-derived renal interstitial and endothelial cells [64••].

However, the problems of connecting the organoids to the host’s urine excretory system and of promoting their maturation so that they can function as adult kidneys are yet to be overcome. Some progress has been made towards connecting transplanted kidney rudiments to the host’s ureter, with one study showing that the ureters of transplanted rat kidney rudiments can be anastomosed to the host’s urinary system [72], and more recently, the group of Yokoo has been able to connect pig kidney rudiment ureters with a bladder generated from a transplanted cloaca [76]. Whilst impressive, these approaches would not be suitable for transplanted organoids as these RPC-derived structures lack a ureter (Fig. 1).

## Decellularization of Kidney Scaffolds

Decellularization of animal or human organs in combination with re-cellularization using autologous progenitor and endothelial cells is the most promising approach to generating bioengineered organs ex vivo and seems to offer the quickest route to clinical applications [77–79]. During the decellularization process, the cellular compartment of a given organ is removed through delivery of a detergent-based solution via the innate vasculature throughout the organ parenchyma. This approach has been successfully used to generate a bioengineered airway consisting of a decellularised cadaveric trachea seeded with autologous MSC-derived chondrocytes and bronchial epithelial cells derived from a patient with bronchial stenosis. The airway was used to replace the stenosed bronchus and the patient had a very good outcome and, importantly, did not require immunosuppressant therapy [80, 81]. In the case of the kidney, a number of decellularization protocols have been established in rodent, pig, rhesus monkey and human kidneys [2••, 82, 83•, 84–87]. These protocols involve the use of detergents or enzymes which are perfused in an antegrade fashion from the renal artery through the kidney vasculature (and sometimes through the ureter) [83•, 85], thus removing all cells [67•, 88, 89]. Importantly, the extracellular matrix (ECM) that remains after the decellularization process maintains the delicate glomerular and tubular structures as well as the vascular tree of the kidney. Furthermore, the ECM is able to modulate the phenotype of seeded progenitor cells, which express renal developmental genes in response [86, 90, 91]. In addition, immunogenicity is reduced since major immunogenicity antigens are lost after decellularization [83•]. This raises the possibility that decellularised kidneys from other species could be used as a source of scaffold for transplantation, the advantage being that such kidneys would be in pristine condition, whereas human kidneys deemed unsuitable for transplantation could have structural damage. The pig is particularly attractive because the size and microarchitecture of pig and human kidneys are similar [92].

Following decellularisation of the kidney scaffold, the next challenge is to repopulate with renal cells and endothelial cells. Most studies have focused on the rat kidney as a model to study the effects of re-cellularisation on cell distribution and function, using various cell combinations, including mouse ESCs [86, 93, 94], human iPSC-derived endothelial cells with human renal cortical tubular epithelial cells [95], rat aorta endothelial with rat epithelial tubular cells [96] and human umbilical vascular endothelial cells with rat neonatal kidney cells [2••]. Other groups have reported re-cellularisation of decellularised mouse, pig and rhesus monkey kidneys using human kidney cells, foetal rhesus monkey kidney cells or ESCs [2••, 82, 87, 94, 97]. Interestingly, ESCs seeded into the kidney scaffolds have been shown to populate the matrix with evidence of site-appropriate differentiation, indicating that the ECM of the decellularised kidneys can instruct the ESCs to differentiate into renal and vascular elements of the kidney [86, 93, 94].

Research in this field is still ongoing, and currently, optimal re-cellularisation techniques are being developed. Three major challenges are being recognised: the requirement for an autologous cell type that can differentiate into both endothelial and specialised kidney cells, a strategy for achieving complete re-cellularisation and the application of a transrenal pressure gradient. Previous studies have explored the use of endothelial and renal progenitor cells from various sources [2••, 95, 96], but it is clear that iPSC-derived RPCs and endothelial cells present the best route forward due to the fact that they are autologous and RPCs can generate all cell types of the nephrons and collecting tubules.

In order to re-cellularise the kidney, Caralt and colleagues performed perfusion experiments separately by injecting either human iPSC-derived endothelial cells or an immortalised human renal cortical tubular epithelial cell line via the renal artery. While excellent vascular repopulation by the endothelial cells was observed, it was found that after 24 h, only 50 % of the renal tubules were re-cellularised by the kidney cells [95]. Using a different approach involving perfusion of rat endothelial cells and tubule epithelial cells via the renal artery (antegrade) and ureter (retrograde), respectively, it was shown that the vascular network could be efficiently repopulated by the endothelial cells, which were able to survive and proliferate, while the tubular epithelial cells failed to populate the kidney scaffold sufficiently. This study also involved the use of a specifically designed bioreactor which allowed the application of a transrenal pressure gradient during the seeding procedure. It was found that the arterial pressure increased in the kidneys repopulated with endothelial cells, indicating functionality of the endothelial cells through changes in flow resistance [96].

The most promising demonstration of re-populating decellularised kidneys has been reported by the Ott group who successfully managed to seed both human umbilical vascular endothelial cells and rat neonatal kidney cells into decellularised rat kidneys. They combined antegrade seeding of endothelial cells with retrograde seeding of the kidney cells under the application of a transrenal pressure gradient in a special bioreactor. The cells populated half of the glomeruli and nephron structures across the kidney scaffold and expressed tissue-specific markers. Furthermore, the repopulated kidneys were assessed for their functionality ex vivo and displayed some degree of filtration capacity, whereas the decellularised kidneys did not. Of note, when the re-cellularised kidneys were transplanted orthotopically into recipient rats, it was found that urine-like solution could be produced, albeit at lower levels than in native kidneys [2••].

Taken together, while highly promising, these results demonstrate that further optimisation is needed to generate fully functional bioengineered kidneys using decellularised scaffolds. Specifically, one of the main challenges is to achieve the full repopulation of the decellularised kidney scaffold in an organotypic way, resulting in correct spatial distribution of appropriately differentiated renal and endothelial cells which physiologically interact to perform the filtration function of the kidney (Fig. 1). As discussed below, this could potentially be overcome by using a bioprinting approach that simultaneously prints the cells along with an appropriate synthetic scaffold, thereby circumventing the repopulation problem.

## 3D Bioprinting

Three-dimensional (3D) bioprinting is an emerging technology that facilitates the layer-by-layer precise positioning of biological materials, biochemicals and living cells, with spatial control of the placement of functional components [98]. 3D printing technology offers alternative approaches to generating organotypic scaffolds for bioengineering of organs. A pioneering study by the group of Atala showed that 3D bioprinting could be used to generate a biodegradable scaffold of a human bladder. These bioengineered bladders were seeded with autologous urothelial and muscle cells and were transplanted into seven patients with non-functional bladders [99]. 3D bioprinting of various other tissues, such as vessels and tracheal grafts, has also been achieved [100, 101]. However, it should be noted that the aforementioned tissues and organs are relatively simple structures, whereas the kidney is much more complex, containing approximately one million nephrons. For this reason, the possibility of engineering a 3D-bioprinted kidney is currently beyond our capabilities. However, with further advances in this technology, it could be possible to combine 3D printing of the kidney scaffold with autologous RPCs and endothelial cells in order to generate personalised kidneys for patients with ESRD (Fig. 1). Interestingly, Organovo Inc. has recently presented a 3D bioprinted model of ‘kidney proximal tubular tissue’. The 3D tissue, which consisted of proximal tubule cells, renal interstitial cells and endothelial networks, could be maintained in culture for up to two weeks [102].

## Safety Issues

The main safety issues concerning bioengineered kidneys relate to the cell types used to repopulate the kidney, and the source of the renal scaffold.

## Cell-Related Safety Issues

As previously mentioned, iPSC-derived RPCs are most promising due to the fact that they can generate all cell types within the kidney [63••, 64••]. However, pluripotent cells such as iPSCs pose particular risks due to their propensity to form teratomas, or even teratocarcinomas. It would therefore be very important to ensure that the RPC population used in the therapy did not contain any undifferentiated iPSCs. Before transplanting bioengineered kidneys into man, it would also be crucial to track their fate in preclinical models in order to assess whether they migrate to distant organs and tissues where they could potentially maldifferentiate or form tumours [103]. Particular caution would be needed with immunosuppressed transplant patients, for it is known that immunosuppressant therapy significantly increases the risk of tumour formation [104]. The use of autologous cells should hopefully obviate the need for immunosuppressants, though they might still be required if the source of scaffold was found to be immunogenic. Despite the concerns related to the use of pluripotent cell therapies, a number of clinical trials are in progress to assess their safety in the treatment of particular diseases, such as the use of ESC-derived retinal pigment epithelial cells in patients with age-related macular degeneration [105]. To date, there have been no reports of recipients of ESC-based therapies developing tumours, which suggest that if sufficient care is taken to ensure that the administered population has a normal karyotype, has been scaled up under conditions of Good Manufacturing Practice, and is not contaminated with undifferentiated cells, the therapy is likely to be safe.

## Scaffold-Related Safety Issues

Decellularised scaffolds are unlikely to pose particular safety issues because they are composed of native ECM. It would be expected that the ECM would be degraded over time and replaced with new ECM derived from the cells used to repopulate the scaffold. A potential safety issue might arise if the rate of ECM degradation was faster than that of new ECM deposition, as this would be expected to affect the integrity of the scaffold. Of note, the patient who received a bioengineered airway generated from a decellularised scaffold remained well at 5 years follow-up [80], suggesting that this could be a safe approach, at least for tissues and organs with simple structures. However, the number of clinical studies conducted to date are too few to confirm the safety and feasibility of this approach [106]. Synthetic and/or bio-printed substrates would be expected to pose more safety issues than bioengineered organs comprising decellularised scaffolds simply because their composition would be different than that of native scaffolds. For this reason, it could be difficult to predict how they might interact with the host over the short or long term. A recent report indicating a high incidence of death (6 out of 8) in patients transplanted with synthetic tracheas [107] demonstrates the need for this field to progress cautiously within a robust regulatory framework.

## Conclusions

In the last few years, tremendous progress has been made towards the development of autologous cells for kidney bioengineering. The key advance has been the development of RPCs derived from pluripotent stem cells (i.e. ESCs and iPSCs) that can generate all cells of the nephron and collecting tubules and have the ability to self-organise in vitro to form renal organoids [63••, 64••]. It is unlikely that these organoids will be useful as a therapy to directly treat patients with ESRD because based on previous studies with rudiment transplants [72], they would probably not mature sufficiently and would not be connected with the host’s urinary excretory system. Nevertheless, renal organoids present an excellent model system for understanding kidney development and disease and for drug screening programmes. In regard to therapy, instead of transplanting renal organoids, a more promising approach would be to use iPSC-derived RPCs and endothelial cells to recellularise kidney scaffolds derived either from human donor kidneys or pig kidneys. Much progress has been made towards this goal [2••], but the key challenge that still needs to be overcome is that of repopulating the nephrons and ensuring that the cells differentiate and function appropriately according to their position along the renal tubule. 3D bioprinting could potentially solve this problem, but despite the significant and exciting advances that have been made, printing complex organs like the kidney will not be happening soon, as the technology requires a considerable amount of time to evolve.

## References

[1] Murray PA, Woolf AS (2014). Using stem and progenitor cells to recapitulate kidney development and restore renal function. Current opinion in organ transplantation.

[2.••] Song JJ, Guyette JP, Gilpin SE, Gonzalez G, Vacanti JP, Ott HC. Regeneration and experimental orthotopic transplantation of a bioengineered kidney. Nature medicine. 2013;19(5):646–51. Pubmed Central PMCID: 3650107. **This study shows that de-cellularised rat kidneys repopulated with neonatal rat kidney and human endothelial cells could display some functionaly following transplantation into adult rats**. 10.1038/nm.3154 PMC3650107 23584091

[3] Al-Awqati Q, Oliver JA (2002). Stem cells in the kidney. Kidney international.

[4] Dekel B, Zangi L, Shezen E, Reich-Zeliger S, Eventov-Friedman S, Katchman H (2006). Isolation and characterization of nontubular sca-1+lin- multipotent stem/progenitor cells from adult mouse kidney. Journal of the American Society of Nephrology : JASN.

[5] Fuente Mora C, Ranghini E, Bruno S, Bussolati B, Camussi G, Wilm B (2012). Differentiation of podocyte and proximal tubule-like cells from a mouse kidney-derived stem cell line. Stem cells and development.

[6] Kitamura S, Yamasaki Y, Kinomura M, Sugaya T, Sugiyama H, Maeshima Y (2005). Establishment and characterization of renal progenitor like cells from S3 segment of nephron in rat adult kidney. FASEB journal : official publication of the Federation of American Societies for Experimental Biology.

[7] Lee PT, Lin HH, Jiang ST, Lu PJ, Chou KJ, Fang HC (2010). Mouse kidney progenitor cells accelerate renal regeneration and prolong survival after ischemic injury. Stem cells.

[8] Oliver JA, Maarouf O, Cheema FH, Martens TP, Al-Awqati Q (2004). The renal papilla is a niche for adult kidney stem cells. The Journal of clinical investigation.

[9] Osafune K, Takasato M, Kispert A, Asashima M, Nishinakamura R (2006). Identification of multipotent progenitors in the embryonic mouse kidney by a novel colony-forming assay. Development.

[10] Presnell SC, Bruce AT, Wallace SM, Choudhury S, Genheimer CW, Cox B (2011). Isolation, characterization, and expansion methods for defined primary renal cell populations from rodent, canine, and human normal and diseased kidneys. Tissue engineering Part C, Methods..

[11] Davies JA, Unbekandt M, Ineson J, Lusis M, Little MH (2012). Dissociation of embryonic kidney followed by re-aggregation as a method for chimeric analysis. Methods in molecular biology..

[12] Ganeva V, Unbekandt M, Davies JA (2011). An improved kidney dissociation and reaggregation culture system results in nephrons arranged organotypically around a single collecting duct system. Organogenesis.

[13] Rak-Raszewska A, Wilm B, Edgar D, Kenny S, Woolf AS, Murray P (2012). Development of embryonic stem cells in recombinant kidneys. Organogenesis.

[14] Unbekandt M, Davies JA (2010). Dissociation of embryonic kidneys followed by reaggregation allows the formation of renal tissues. Kidney international.

[15] Ranghini E, Fuente Mora C, Edgar D, Kenny SE, Murray P, Wilm B (2013). Stem cells derived from neonatal mouse kidney generate functional proximal tubule-like cells and integrate into developing nephrons in vitro. PloS one.

[16] Buzhor E, Omer D, Harari-Steinberg O, Dotan Z, Vax E, Pri-Chen S (2013). Reactivation of NCAM1 defines a subpopulation of human adult kidney epithelial cells with clonogenic and stem/progenitor properties. The American journal of pathology.

[17] Fesenko I, Franklin D, Garnett P, Bass P, Campbell S, Hardyman M (2010). Stem cell marker TRA-1-60 is expressed in foetal and adult kidney and upregulated in tubulo-interstitial disease. Histochemistry and cell biology.

[18] Bussolati B, Bruno S, Grange C, Buttiglieri S, Deregibus MC, Cantino D (2005). Isolation of renal progenitor cells from adult human kidney. The American journal of pathology.

[19] Bussolati B, Moggio A, Collino F, Aghemo G, D’Armento G, Grange C (2012). Hypoxia modulates the undifferentiated phenotype of human renal inner medullary CD133+ progenitors through Oct4/miR-145 balance. American journal of physiology Renal physiology.

[20] Sagrinati C, Netti GS, Mazzinghi B, Lazzeri E, Liotta F, Frosali F (2006). Isolation and characterization of multipotent progenitor cells from the Bowman’s capsule of adult human kidneys. Journal of the American Society of Nephrology : JASN.

[21] Ward HH, Romero E, Welford A, Pickett G, Bacallao R, Gattone VH (2011). Adult human CD133/1(+) kidney cells isolated from papilla integrate into developing kidney tubules. Biochimica et biophysica acta.

[22] Grange C, Moggio A, Tapparo M, Porta S, Camussi G, Bussolati B (2014). Protective effect and localization by optical imaging of human renal CD133+ progenitor cells in an acute kidney injury model. Physiological reports.

[23] Ronconi E, Sagrinati C, Angelotti ML, Lazzeri E, Mazzinghi B, Ballerini L (2009). Regeneration of glomerular podocytes by human renal progenitors. Journal of the American Society of Nephrology : JASN.

[24] Guimaraes-Souza NK, Yamaleyeva LM, AbouShwareb T, Atala A, Yoo JJ (2012). In vitro reconstitution of human kidney structures for renal cell therapy. Nephrology, dialysis, transplantation : official publication of the European Dialysis and Transplant Association - European Renal Association..

[25] Kim IH, Ko IK, Atala A, Yoo JJ (2015). Whole kidney engineering for clinical translation. Current opinion in organ transplantation.

[26] Morigi M, Imberti B, Zoja C, Corna D, Tomasoni S, Abbate M (2004). Mesenchymal stem cells are renotropic, helping to repair the kidney and improve function in acute renal failure. Journal of the American Society of Nephrology : JASN.

[27] Yokoo T, Ohashi T, Shen JS, Sakurai K, Miyazaki Y, Utsunomiya Y (2005). Human mesenchymal stem cells in rodent whole-embryo culture are reprogrammed to contribute to kidney tissues. Proceedings of the National Academy of Sciences of the United States of America.

[28] Bi B, Schmitt R, Israilova M, Nishio H, Cantley LG (2007). Stromal cells protect against acute tubular injury via an endocrine effect. Journal of the American Society of Nephrology : JASN.

[29] Collino F, Bruno S, Incarnato D, Dettori D, Neri F, Provero P (2015). AKI recovery induced by mesenchymal stromal cell-derived extracellular vesicles carrying MicroRNAs. Journal of the American Society of Nephrology : JASN.

[30] Gatti S, Bruno S, Deregibus MC, Sordi A, Cantaluppi V, Tetta C (2011). Microvesicles derived from human adult mesenchymal stem cells protect against ischaemia-reperfusion-induced acute and chronic kidney injury. Nephrology, dialysis, transplantation : official publication of the European Dialysis and Transplant Association - European Renal Association..

[31.•] Geng Y, Zhang L, Fu B, Zhang J, Hong Q, Hu J, et al. Mesenchymal stem cells ameliorate rhabdomyolysis-induced acute kidney injury via the activation of M2 macrophages. Stem cell research & therapy. 2014;5(3):80. Pubmed Central PMCID: 4230233. **This work shows that MSCs injected intravenously can ameliorate renal injury despite being entrapped in the pulmonary vasculature**. 10.1186/scrt469 PMC4230233 24961539

[32] Togel F, Hu Z, Weiss K, Isaac J, Lange C, Westenfelder C (2005). Administered mesenchymal stem cells protect against ischemic acute renal failure through differentiation-independent mechanisms. American journal of physiology Renal physiology.

[33] Togel F, Weiss K, Yang Y, Hu Z, Zhang P, Westenfelder C (2007). Vasculotropic, paracrine actions of infused mesenchymal stem cells are important to the recovery from acute kidney injury. American journal of physiology Renal physiology.

[34] Donizetti-Oliveira C, Semedo P, Burgos-Silva M, Cenedeze MA, Malheiros DM, Reis MA (2012). Adipose tissue-derived stem cell treatment prevents renal disease progression. Cell transplantation.

[35] Bai ZM, Deng XD, Li JD, Li DH, Cao H, Liu ZX (2013). Arterially transplanted mesenchymal stem cells in a mouse reversible unilateral ureteral obstruction model: in vivo bioluminescence imaging and effects on renal fibrosis. Chinese medical journal.

[36] Baulier E, Favreau F, Le Corf A, Jayle C, Schneider F, Goujon JM (2014). Amniotic fluid-derived mesenchymal stem cells prevent fibrosis and preserve renal function in a preclinical porcine model of kidney transplantation. Stem cells translational medicine.

[37] Togel F, Yang Y, Zhang P, Hu Z, Westenfelder C (2008). Bioluminescence imaging to monitor the in vivo distribution of administered mesenchymal stem cells in acute kidney injury. American journal of physiology Renal physiology.

[38] Rowart P, Erpicum P, Detry O, Weekers L, Gregoire C, Lechanteur C (2015). Mesenchymal stromal cell therapy in ischemia/reperfusion injury. Journal of immunology research..

[39] Kunter U, Rong S, Boor P, Eitner F, Muller-Newen G, Djuric Z (2007). Mesenchymal stem cells prevent progressive experimental renal failure but maldifferentiate into glomerular adipocytes. Journal of the American Society of Nephrology : JASN.

[40] Feng Z, Ting J, Alfonso Z, Strem BM, Fraser JK, Rutenberg J (2010). Fresh and cryopreserved, uncultured adipose tissue-derived stem and regenerative cells ameliorate ischemia-reperfusion-induced acute kidney injury. Nephrology, dialysis, transplantation : official publication of the European Dialysis and Transplant Association - European Renal Association..

[41] Gimble JM, Guilak F, Bunnell BA (2010). Clinical and preclinical translation of cell-based therapies using adipose tissue-derived cells. Stem cell research & therapy.

[42] Katsuno T, Ozaki T, Saka Y, Furuhashi K, Kim H, Yasuda K (2013). Low serum cultured adipose tissue-derived stromal cells ameliorate acute kidney injury in rats. Cell transplantation.

[43] Perin EC, Sanz-Ruiz R, Sanchez PL, Lasso J, Perez-Cano R, Alonso-Farto JC (2014). Adipose-derived regenerative cells in patients with ischemic cardiomyopathy: the PRECISE Trial. American heart journal.

[44] Wankhade UD, Shen M, Kolhe R, Fulzele S (2016). Advances in adipose-derived stem cells isolation, characterization, and application in regenerative tissue engineering. Stem cells international..

[45] Fraser JK, Hicok KC, Shanahan R, Zhu M, Miller S, Arm DM (2014). The celution system: automated processing of adipose-derived regenerative cells in a functionally closed system. Advances in wound care.

[46] Hicok KC, Hedrick MH (2011). Automated isolation and processing of adipose-derived stem and regenerative cells. Methods in molecular biology..

[47] Baer PC, Bereiter-Hahn J, Missler C, Brzoska M, Schubert R, Gauer S (2009). Conditioned medium from renal tubular epithelial cells initiates differentiation of human mesenchymal stem cells. Cell proliferation.

[48] Singaravelu K, Padanilam BJ (2009). In vitro differentiation of MSC into cells with a renal tubular epithelial-like phenotype. Renal failure.

[49] Kuzma-Kuzniarska M, Rak-Raszewska A, Kenny S, Edgar D, Wilm B, Fuente Mora C (2012). Integration potential of mouse and human bone marrow-derived mesenchymal stem cells. Differentiation; research in biological diversity.

[50] Kim D, Dressler GR (2005). Nephrogenic factors promote differentiation of mouse embryonic stem cells into renal epithelia. Journal of the American Society of Nephrology : JASN.

[51] Kobayashi T, Tanaka H, Kuwana H, Inoshita S, Teraoka H, Sasaki S (2005). Wnt4-transformed mouse embryonic stem cells differentiate into renal tubular cells. Biochemical and biophysical research communications.

[52] Steenhard BM, Isom KS, Cazcarro P, Dunmore JH, Godwin AR, St John PL (2005). Integration of embryonic stem cells in metanephric kidney organ culture. Journal of the American Society of Nephrology : JASN.

[53] Vigneau C, Polgar K, Striker G, Elliott J, Hyink D, Weber O (2007). Mouse embryonic stem cell-derived embryoid bodies generate progenitors that integrate long term into renal proximal tubules in vivo. Journal of the American Society of Nephrology : JASN.

[54] Takahashi K, Tanabe K, Ohnuki M, Narita M, Ichisaka T, Tomoda K (2007). Induction of pluripotent stem cells from adult human fibroblasts by defined factors. Cell.

[55] Gonzalez F, Boue S, Izpisua Belmonte JC (2011). Methods for making induced pluripotent stem cells: reprogramming a la carte. Nature reviews Genetics.

[56] Schlaeger TM, Daheron L, Brickler TR, Entwisle S, Chan K, Cianci A (2015). A comparison of non-integrating reprogramming methods. Nature biotechnology.

[57] Fan H, Johnson C (2011). Insertional oncogenesis by non-acute retroviruses: implications for gene therapy. Viruses.

[58.••] Taguchi A, Kaku Y, Ohmori T, Sharmin S, Ogawa M, Sasaki H (2014). Redefining the in vivo origin of metanephric nephron progenitors enables generation of complex kidney structures from pluripotent stem cells. Cell stem cell.

[59] Araoka T, Mae S, Kurose Y, Uesugi M, Ohta A, Yamanaka S (2014). Efficient and rapid induction of human iPSCs/ESCs into nephrogenic intermediate mesoderm using small molecule-based differentiation methods. PloS one.

[60] Imberti B, Tomasoni S, Ciampi O, Pezzotta A, Derosas M, Xinaris C (2015). Renal progenitors derived from human iPSCs engraft and restore function in a mouse model of acute kidney injury. Scientific reports..

[61] Lam AQ, Freedman BS, Morizane R, Lerou PH, Valerius MT, Bonventre JV (2014). Rapid and efficient differentiation of human pluripotent stem cells into intermediate mesoderm that forms tubules expressing kidney proximal tubular markers. Journal of the American Society of Nephrology : JASN.

[62] Mae S, Shono A, Shiota F, Yasuno T, Kajiwara M, Gotoda-Nishimura N (2013). Monitoring and robust induction of nephrogenic intermediate mesoderm from human pluripotent stem cells. Nature communications..

[63.••] Morizane R, Lam AQ, Freedman BS, Kishi S, Valerius MT, Bonventre JV (2015). Nephron organoids derived from human pluripotent stem cells model kidney development and injury. Nature biotechnology.

[64.••] Takasato M, Er PX, Chiu HS, Maier B, Baillie GJ, Ferguson C (2015). Kidney organoids from human iPS cells contain multiple lineages and model human nephrogenesis. Nature.

[65] Xia Y, Nivet E, Sancho-Martinez I, Gallegos T, Suzuki K, Okamura D (2013). Directed differentiation of human pluripotent cells to ureteric bud kidney progenitor-like cells. Nature cell biology.

[66] Xia Y, Sancho-Martinez I, Nivet E, Rodriguez Esteban C, Campistol JM, Izpisua Belmonte JC (2014). The generation of kidney organoids by differentiation of human pluripotent cells to ureteric bud progenitor-like cells. Nature protocols.

[67.•] Montserrat N, Garreta E, Izpisua Belmonte JC. Regenerative strategies for kidney engineering. FEBS J. 2016. **This comprehensive review discusses the development of protocols for directing the differentiation of pluripotent stem cells to the renal lineage along with recent advances in kidney bioengineering**. 10.1111/febs.13704 26938311

[68] Freedman BS, Brooks CR, Lam AQ, Fu H, Morizane R, Agrawal V (2015). Modelling kidney disease with CRISPR-mutant kidney organoids derived from human pluripotent epiblast spheroids. Nature communications..

[69] Dekel B, Burakova T, Arditti FD, Reich-Zeliger S, Milstein O, Aviel-Ronen S (2003). Human and porcine early kidney precursors as a new source for transplantation. Nature medicine.

[70] Rogers SA, Lowell JA, Hammerman NA, Hammerman MR (1998). Transplantation of developing metanephroi into adult rats. Kidney international.

[71] Woolf AS, Palmer SJ, Snow ML, Fine LG (1990). Creation of a functioning chimeric mammalian kidney. Kidney international.

[72] Dilworth MR, Clancy MJ, Marshall D, Bravery CA, Brenchley PE, Ashton N (2008). Development and functional capacity of transplanted rat metanephroi. Nephrology, dialysis, transplantation : official publication of the European Dialysis and Transplant Association - European Renal Association..

[73] Matsumoto K, Yokoo T, Matsunari H, Iwai S, Yokote S, Teratani T (2012). Xenotransplanted embryonic kidney provides a niche for endogenous mesenchymal stem cell differentiation into erythropoietin-producing tissue. Stem cells.

[74] Matsumoto K, Yokoo T, Yokote S, Utsunomiya Y, Ohashi T, Hosoya T (2012). Functional development of a transplanted embryonic kidney: effect of transplantation site. Journal of nephrology.

[75] Wilm B, Murray P (2015). Amniotic fluid stem cells within chimeric kidney rudiments differentiate to functional podocytes after transplantation into mature rat kidneys.

[76] Yokote S, Matsunari H, Iwai S, Yamanaka S, Uchikura A, Fujimoto E (2015). Urine excretion strategy for stem cell-generated embryonic kidneys. Proceedings of the National Academy of Sciences of the United States of America.

[77] Badylak SF, Taylor D, Uygun K (2011). Whole-organ tissue engineering: decellularization and recellularization of three-dimensional matrix scaffolds. Annual review of biomedical engineering..

[78] Badylak SF, Weiss DJ, Caplan A, Macchiarini P (2012). Engineered whole organs and complex tissues. Lancet.

[79] Orlando G, Soker S, Stratta RJ (2013). Organ bioengineering and regeneration as the new Holy Grail for organ transplantation. Annals of surgery.

[80] Gonfiotti A, Jaus MO, Barale D, Baiguera S, Comin C, Lavorini F (2014). The first tissue-engineered airway transplantation: 5-year follow-up results. Lancet.

[81] Macchiarini P, Jungebluth P, Go T, Asnaghi MA, Rees LE, Cogan TA (2008). Clinical transplantation of a tissue-engineered airway. Lancet.

[82] Nakayama KH, Batchelder CA, Lee CI, Tarantal AF (2010). Decellularized rhesus monkey kidney as a three-dimensional scaffold for renal tissue engineering. Tissue engineering Part A.

[83.•] Orlando G, Booth C, Wang Z, Totonelli G, Ross CL, Moran E (2013). Discarded human kidneys as a source of ECM scaffold for kidney regeneration technologies. Biomaterials.

[84] Orlando G, Farney AC, Iskandar SS, Mirmalek-Sani SH, Sullivan DC, Moran E (2012). Production and implantation of renal extracellular matrix scaffolds from porcine kidneys as a platform for renal bioengineering investigations. Annals of surgery.

[85] Peloso A, Petrosyan A, Da Sacco S, Booth C, Zambon JP, O’Brien T (2015). Renal extracellular matrix scaffolds from discarded kidneys maintain glomerular morphometry and vascular resilience and retains critical growth factors. Transplantation.

[86] Ross EA, Williams MJ, Hamazaki T, Terada N, Clapp WL, Adin C (2009). Embryonic stem cells proliferate and differentiate when seeded into kidney scaffolds. Journal of the American Society of Nephrology : JASN.

[87] Sullivan DC, Mirmalek-Sani SH, Deegan DB, Baptista PM, Aboushwareb T, Atala A (2012). Decellularization methods of porcine kidneys for whole organ engineering using a high-throughput system. Biomaterials.

[88] Petrosyan A, Zanusso I, Lavarreda-Pearce M, Leslie S, Sedrakyan S, De Filippo RE (2016). Decellularized renal matrix and regenerative medicine of the kidney: a different point of view.

[89] Scarritt ME, Pashos NC, Bunnell BA (2015). A review of cellularization strategies for tissue engineering of whole organs. Frontiers in bioengineering and biotechnology..

[90] Nakayama KH, Lee CC, Batchelder CA, Tarantal AF (2013). Tissue specificity of decellularized rhesus monkey kidney and lung scaffolds. PloS one.

[91] Petrosyan A, Orlando G, Peloso A, Wang Z, Farney AC, Rogers J (2015). Understanding the bioactivity of stem cells seeded on extracellular matrix scaffolds produced from discarded human kidneys: a critical step towards a new generation bio-artificial kidney. CellR4.

[92] Sampaio FJ, Pereira-Sampaio MA, Favorito LA (1998). The pig kidney as an endourologic model: anatomic contribution. J Endourol.

[93] Bonandrini B, Figliuzzi M, Papadimou E, Morigi M, Perico N, Casiraghi F (2014). Recellularization of well-preserved acellular kidney scaffold using embryonic stem cells. Tissue engineering Part A.

[94] Guan Y, Liu S, Sun C, Cheng G, Kong F, Luan Y (2015). The effective bioengineering method of implantation decellularized renal extracellular matrix scaffolds. Oncotarget.

[95] Caralt M, Uzarski JS, Iacob S, Obergfell KP, Berg N, Bijonowski BM (2015). Optimization and critical evaluation of decellularization strategies to develop renal extracellular matrix scaffolds as biological templates for organ engineering and transplantation. American journal of transplantation : official journal of the American Society of Transplantation and the American Society of Transplant Surgeons.

[96] Hachisuka S, Sato Y, Yoshiike M, Nakazawa R, Sasaki H, Chikaraishi T (2015). Enhanced recellularization of renal extracellular matrix scaffold under negative pressure. Integr Mol Med.

[97] Batchelder CA, Martinez ML, Tarantal AF (2015). Natural scaffolds for renal differentiation of human embryonic stem cells for kidney tissue engineering. PloS one.

[98] Peloso A, Katari R, Murphy SV, Zambon JP, DeFrancesco A, Farney AC (2015). Prospect for kidney bioengineering: shortcomings of the status quo. Expert Opin Biol Ther.

[99] Atala A, Bauer SB, Soker S, Yoo JJ, Retik AB (2006). Tissue-engineered autologous bladders for patients needing cystoplasty. Lancet.

[100] Visconti RP, Kasyanov V, Gentile C, Zhang J, Markwald RR, Mironov V (2010). Towards organ printing: engineering an intra-organ branched vascular tree. Expert Opin Biol Ther.

[101] Chang JW, Park SA, Park JK, Choi JW, Kim YS, Shin YS (2014). Tissue-engineered tracheal reconstruction using three-dimensionally printed artificial tracheal graft: preliminary report. Artificial organs.

[102] King S, Creasey O, Presnell S, Nguyen D (2015). Design and characterization of a multicellular, three-dimensional (3D) tissue model of the human kidney proximal tubule. FASEB journal : official publication of the Federation of American Societies for Experimental Biology.

[103] Heslop JA, Hammond TG, Santeramo I, Tort Piella A, Hopp I, Zhou J (2015). Concise review: workshop review: understanding and assessing the risks of stem cell-based therapies. Stem cells translational medicine.

[104] Gallagher MP, Kelly PJ, Jardine M, Perkovic V, Cass A, Craig JC (2010). Long-term cancer risk of immunosuppressive regimens after kidney transplantation. Journal of the American Society of Nephrology : JASN.

[105] Whiting P, Kerby J, Coffey P, da Cruz L, McKernan R (2015). Progressing a human embryonic stem-cell-based regenerative medicine therapy towards the clinic. Philosophical transactions of the Royal Society of London Series B, Biological sciences.

[106] Baiguera S, Urbani L, Del Gaudio C (2014). Tissue engineered scaffolds for an effective healing and regeneration: reviewing orthotopic studies. BioMed research international..

[107] Cyranoski D (2014). Investigations launched into artificial tracheas. Nature.

